# Supplementary material for: To help or punish in the face of unfairness: men and women prefer mutually-beneficial strategies over punishment in a sexual selection context
Source: R Soc Open Sci. 2019 Sep 4;6(9):181441. doi: 10.1098/rsos.181441 (PMC6774947; doi:10.1098/rsos.181441)
Supplement: Supplementary File For “To Help or Punish in the Face of Unfairness: Men and Women Prefer Mutually-Beneficial Strategies over Punishment in a Sexual Selection Context” authored by Ferguson et al and published in Royal Society Open Science [file rsos181441supp1.docx]

**Supplementary File For “To Help or Punish in the Face of Unfairness: Men and Women Prefer Mutually-Beneficial Strategies over Punishment in a Sexual Selection Context” authored by Eamonn Ferguson, Erin Quigley, Georgia Powell, Liam Stewart, Frey Harrison & Holly Tallentire for Royal Society Open Science**

| **File** | **Pages** |
| --- | --- |
| *Section A: Materials for Studies 1, 2 and 3* | 2-20 |
| *Section B: Equivalence of Pure Control and Active Control for Priming* | 21-23 |
| *Section C: Interpretation of Attractiveness Ratings for Study Two* | 24 |
| *Section D: Additional Analyses for Studies One, Two and Three*  *Section E: Details for GEE Models*  *Section F: Syntax for analyses linked to file Doi:* <https://datadryad.org/review?doi=doi:10.5061/dryad.738pm17> | 25-39  40-40  41-58 |

**Section A: Materials for Study 1, 2 and 3**

This file contains the materials in [A] study one, [B] study two and [C] study three and [D] Cultural context of the studies

**[1] Study 1: Instructions and Materials**

**Romantic Prime:** Please write down the number of the female you are most attracted to, and then write a brief summary of your ideal date with her. You may have up to 3 minutes to think about and write a brief description.

**Control Prime:** Please choose one of the road scenes above. Which one did you choose_____? Please imagine being on that street. Please write about the most pleasant weather conditions in which to walk around and look at the buildings on that street. You may have up to 3 minutes to think about and write a brief description.”

**Punishment and Compensation game**

In the following you will be given a description of a simple game involving the exchange of money. The money you play with is real. At the end of the whole experiment 5 participants will be randomly selected and they will paid based on their decision in the task below. Whatever money you decide to keep you will get. So treat this as a real money task. We will ask you to leave an email address at the end so we can contact you to pay you if you are selected.

In this game there are 3 players: A, B and C.

**Player A** has £10 which they have been given. Player A can decide to give Player B (who they do not know and will never meet) some, none or all of the £10.

**Player B** has £0. Player B has to accept whatever amount of money Player A decides to give them.

**Player C** has £5. Player C can choose to spend some of that £5 to either *compensate player B* or *punish player A* or *do a mixture of compensate and punishment*. Every £1 player C spends to compensate player B or punish player A will result in player B gaining £2 and player A losing £2.

**The Diagram below is a schematic of the game**

**
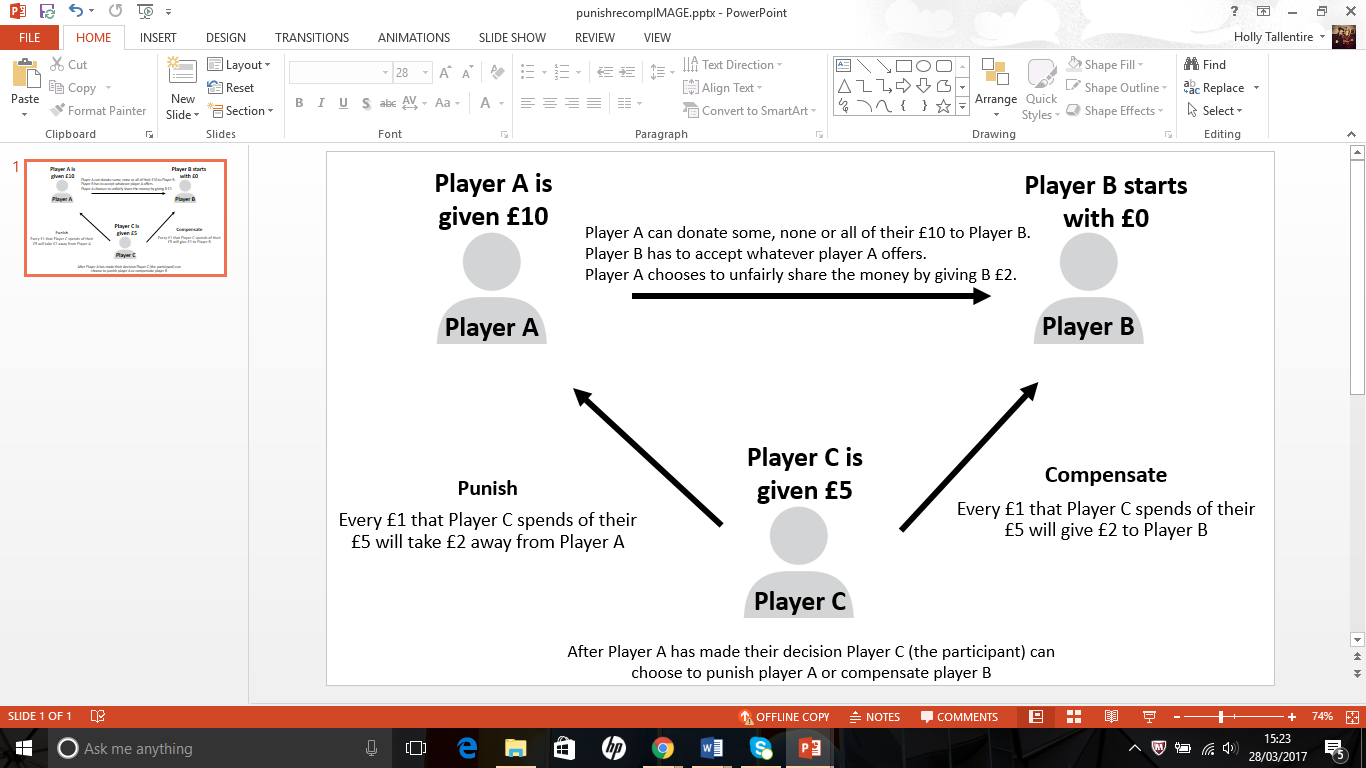
**

For example, if player A gives player B £4 then player A will have £6 of their £10 left. Player B now has £4. Player C can choose to

1. **Do nothing** and keep their £5
2. **Compensate player B.**

For example spend £1 of their money. Now player A will have £6, player B £6 and player C have £4

1. **Punish player A**.

For example spend £1 of their money. Now player A will have £4, player B £4 and player C have £4

1. **Compensate player B and punish player A**.

For example spend £1 of their money to compensate player B and £1 to punish player A. Now player A will have £4, player B £6 and player C have £3.

Just to check you understand the game, can you answer the following questions

If player A gives player B £1, if player C then…

1. Does nothing.

How much money do player A have ______, Player B______ and Player C______

1. Compensates player B by spending £1 of their money.

How much money do player A have ______, Player B______ and Player C______

1. Punish player A by spend £1 of their money.

How much money do player A have ______, Player B______ and Player C______

1. Compensate player B and punish player A by spend £1 of their money to re-compensate player B and £1 to punish player A.

How much money do player A have ______, Player B______ and Player C______

**Now for the real game**

In this game imagine you have been randomly allocated to be player C.

And imagine that player A has decided to give Player B £2.

As player C which of the following would you do (choice 1, 2, 3, or 4 below):

1. **Do nothing**? Y / N
2. **Compensate Player B** Y / N

If yes how much? ________

1. **Punish Player A?** Y / N

If yes how much? ________

1. **Compensate Player B AND punish Player A** Y / N

If yes how much: Compensate player B? ________ Punish Player A?________

If you want to be potentially selected and paid please leave your email below

University Email________________________________________

**Study 1 Post Game Measures**

Considering the behaviour of Player A, to what extent did it make you feel?

|  | **Not at all** | |  |  |  | **Extremely** | |
| --- | --- | --- | --- | --- | --- | --- | --- |
| Uneasy | 1 | 2 | 3 | 4 | 5 | 6 | 7 |
| Worried | 1 | 2 | 3 | 4 | 5 | 6 | 7 |
| Soft-hearted | 1 | 2 | 3 | 4 | 5 | 6 | 7 |
| Sad | 1 | 2 | 3 | 4 | 5 | 6 | 7 |
| Alarmed | 1 | 2 | 3 | 4 | 5 | 6 | 7 |
| Compassionate | 1 | 2 | 3 | 4 | 5 | 6 | 7 |
| Heavy Hearted | 1 | 2 | 3 | 4 | 5 | 6 | 7 |
| Sympathetic | 1 | 2 | 3 | 4 | 5 | 6 | 7 |
| Low spirited | 1 | 2 | 3 | 4 | 5 | 6 | 7 |
| Tender | 1 | 2 | 3 | 4 | 5 | 6 | 7 |
| Irritated | 1 | 2 | 3 | 4 | 5 | 6 | 7 |
| Angry | 1 | 2 | 3 | 4 | 5 | 6 | 7 |
| Upset | 1 | 2 | 3 | 4 | 5 | 6 | 7 |
| Annoyed | 1 | 2 | 3 | 4 | 5 | 6 | 7 |
| Offended | 1 | 2 | 3 | 4 | 5 | 6 | 7 |
| Outraged | 1 | 2 | 3 | 4 | 5 | 6 | 7 |
| Mad | 1 | 2 | 3 | 4 | 5 | 6 | 7 |
| Frustrated | 1 | 2 | 3 | 4 | 5 | 6 | 7 |
| Perturbed | 1 | 2 | 3 | 4 | 5 | 6 | 7 |
| Pleased | 1 | 2 | 3 | 4 | 5 | 6 | 7 |
| Satisfied | 1 | 2 | 3 | 4 | 5 | 6 | 7 |
| Lucky | 1 | 2 | 3 | 4 | 5 | 6 | 7 |
| Content | 1 | 2 | 3 | 4 | 5 | 6 | 7 |

To index emotions of moral outrage, empathic concern, empathic distress and sadness towards Player 1a

***Moral Out-Rage from***

Batson CD, Kennedy CL, Nord L, Stocks EL, Fleming DA, Marzette CM, Lishner DA, Hayes RE, Kolchinsky LM, Zerger T. (2007). Anger at unfairness: Is it moral outrage? Euro J Soc Psychol, **37***,* 1272-1285 (doi 10.1001/ejsp.434).

Empathic concern, empathic distress and sadness from

Cialdini RB, Brown SL, Lewis BP, Luce C, Neuberg SL. (1997). Reinterpreting the empathy-altruism relationship: when one into one equals oneness. J Pers Soc Psychol, **73**, 481-494.(doi. 0022-3514/97/53.00)

The following, are questions about your personal background. You should attempt to fill in all the answers. However, if you prefer not to, or cannot answer a particular question, please leave it blank.

1. What is your sex? **F / M**
2. How old are you? ____________
3. What course do you study? ____________
4. What year of your course are you in? ____________
5. What is your ethnicity? ____________
6. What is your religion? ____________
   1. If you do have a religion, is your religious

attendance regular (i.e. once/week) ? **Y / N**

1. What is your relationship status?

Single

In a monogamous relationship (one sexual partner)

In a polygamous relationship (more than one sexual partner)

Engaged

Married

Separated

Divorced

Widowed

If single, how long has it been since your last relationship? _____________

1. How many brother or sisters do you have? _____________

If you have brothers or sisters, are you the

oldest/youngest/middle/others? _____________

1. Are your close family members involved in any form

of charity work? (e.g. fund-raising) **Y / N**

1. Have you ever considered giving up your time to help **Y / N**

others by volunteering for charitable work?

1. Have you ever done community/charity work? **Y / N**

**How many days in the last year?**

*please tick appropriately*

a. I have taken part in fund-raising events.

1. I have donated blood.
2. I have helped in an old folks home.
3. I have helped in a children’s home.
4. Donated money
5. Are you on the organ donor register
6. Helped in a hospital
7. Others (please specify)
8. Would you donate money to charity if:
   1. It came from your salary
   2. Personal donations from savings
   3. You gave to a registered charity street collector
   4. You had to go to a post/office or bank to do so
   5. Would you like to give directly to a person

such as a homeless person on the street

| **I would/do volunteer to help others because ...** |  |  |  |  |  |  |  |
| --- | --- | --- | --- | --- | --- | --- | --- |
|  | Strongly Disagree |  |  |  |  |  | Strongly Agree |
| 1. I cannot trust others to help | 1 | 2 | 3 | 4 | 5 | 6 | 7 |
| 1. very few people help others these days | 1 | 2 | 3 | 4 | 5 | 6 | 7 |
| 1. I get frustrated that other people do not help those in need | 1 | 2 | 3 | 4 | 5 | 6 | 7 |
| 1. I get angry because others do not help those in need | 1 | 2 | 3 | 4 | 5 | 6 | 7 |
| 1. I am ashamed that other people do not help others | 1 | 2 | 3 | 4 | 5 | 6 | 7 |
| 1. I get upset by people’s disregard of others in need | 1 | 2 | 3 | 4 | 5 | 6 | 7 |
| 1. I feel sad that people generally do not help each others | 1 | 2 | 3 | 4 | 5 | 6 | 7 |
| 1. I feel our society is generally uncaring | 1 | 2 | 3 | 4 | 5 | 6 | 7 |
| 1. many people are only interested in themselves these days | 1 | 2 | 3 | 4 | 5 | 6 | 7 |
| 1. reluctantly many people do not want to help others | 1 | 2 | 3 | 4 | 5 | 6 | 7 |
| 1. most people are selfish | 1 | 2 | 3 | 4 | 5 | 6 | 7 |
| 1. it seems that it is culturally acceptable these days to put self before others | 1 | 2 | 3 | 4 | 5 | 6 | 7 |
| 1. people these days consider their own needs before others | 1 | 2 | 3 | 4 | 5 | 6 | 7 |
| 1. we live in a selfish society | 1 | 2 | 3 | 4 | 5 | 6 | 7 |

Scale to assess reluctant altruism. This is a scale that is currently under construction and not analysed in relation to this paper.

The following statements inquire about your thoughts and feelings in a variety of situations. For each item, indicate how well it describes you by circling the appropriate number on the scale. READ EACH ITEM CAREFULLY BEFORE RESPONDING. Answer as honestly as you can.

Thank you.

***1. Does not describe me very well***

***2. Describes me only moderately well***

***3. Neither inaccurate nor accurate***

***4. Describes me fairly well***

***5. Describes me very well***

|  | ***Does not describe me very well*** |  |  |  | ***Describes me very well*** |
| --- | --- | --- | --- | --- | --- |
| 1. If I see that someone is feeling mad because he or she was mistreated, then I feel mad too. | 1 | 2 | 3 | 4 | 5 |
| 2. When I see someone feeling sad because he or she was hurt by another person, I feel angry. | 1 | 2 | 3 | 4 | 5 |
| 3. I feel angry for other people when they have been victimized by others. | 1 | 2 | 3 | 4 | 5 |
| 4. I feel angry for a person when his or her feelings have been hurt by someone else. | 1 | 2 | 3 | 4 | 5 |
| 5. I get angry when a friend of mine is hurt by someone else. | 1 | 2 | 3 | 4 | 5 |
| 6. When someone I know gets angry at someone else, I feel angry at that person too. | 1 | 2 | 3 | 4 | 5 |
| 7. When I see others being taken advantage of, I don’t feel mad for them. | 1 | 2 | 3 | 4 | 5 |

The Trait Empathic Anger Scale used to index empathic anger analysed in this paper.

Vitaglioe GD, Barnett MA. (2003). Assessing a new dimension of empathy: Empathic anger as a predictor of helping and punishing desires. Mot Emot, **27**, 301-325. (Doi 0146-7239/1200-0301/0)

|  | ***Does not describe me very well*** |  |  |  | ***Describes me very well*** |
| --- | --- | --- | --- | --- | --- |
| 1. I daydream and fantasize, with some regularity, about things that might happen to me. | 1 | 2 | 3 | 4 | 5 |
| 2. I often have tender, concerned feelings for people less fortunate than me. | 1 | 2 | 3 | 4 | 5 |
| 3. I sometimes find it difficult to see things from the "other guy's" point of view. | 1 | 2 | 3 | 4 | 5 |
| 4. Sometimes I don't feel very sorry for other people when they are having problems. | 1 | 2 | 3 | 4 | 5 |
|  |  |  |  |  |  |
| 5. I really get involved with the feelings of the characters in a novel. | 1 | 2 | 3 | 4 | 5 |
| 6. In emergency situations, I feel apprehensive and ill-at-ease. | 1 | 2 | 3 | 4 | 5 |
| 7. I am usually objective when I watch a movie or play, and I don't often get completely caught up in it. | 1 | 2 | 3 | 4 | 5 |
| 8. I try to look at everybody's side of a disagreement before I make a decision. | 1 | 2 | 3 | 4 | 5 |
| 9. When I see someone being taken advantage of, I feel kind of protective towards them. | 1 | 2 | 3 | 4 | 5 |
| 10. I sometimes feel helpless when I am in the middle of a very emotional situation. | 1 | 2 | 3 | 4 | 5 |
| 11. I sometimes try to understand my friends better by imagining how things look from their perspective. | 1 | 2 | 3 | 4 | 5 |
| 12. Becoming extremely involved in a good book or movie is somewhat rare for me. | 1 | 2 | 3 | 4 | 5 |
| 13. When I see someone get hurt, I tend to remain calm. | 1 | 2 | 3 | 4 | 5 |
| 14. Other people's misfortunes do not usually disturb me a great deal. | 1 | 2 | 3 | 4 | 5 |
| 15. If I'm sure I'm right about something, I don't waste much time listening to other people's arguments. | 1 | 2 | 3 | 4 | 5 |
| 16. After seeing a play or movie, I have felt as though I were one of the characters. | 1 | 2 | 3 | 4 | 5 |
|  |  |  |  |  |  |
| 17. Being in a tense emotional situation scares me. | 1 | 2 | 3 | 4 | 5 |
| 18. When I see someone being treated unfairly, I sometimes don't feel very much pity for them. | 1 | 2 | 3 | 4 | 5 |
| 19. I am usually pretty effective in dealing with emergencies. | 1 | 2 | 3 | 4 | 5 |
| 20. I am often quite touched by things that I see happen. | 1 | 2 | 3 | 4 | 5 |
| 21. I believe that there are two sides to every question and try to look at them both. | 1 | 2 | 3 | 4 | 5 |
| 22. I would describe myself as a pretty soft-hearted person. | 1 | 2 | 3 | 4 | 5 |
| 23. When I watch a good movie, I can very easily put myself in the place of a leading character. | 1 | 2 | 3 | 4 | 5 |
| 24. I tend to lose control during emergencies. | 1 | 2 | 3 | 4 | 5 |
| 25. When I'm upset at someone, I usually try to "put myself in his shoes" for a while. | 1 | 2 | 3 | 4 | 5 |
| 26. When I am reading an interesting story or novel, I imagine how I would feel if the events in the story were happening to me. | 1 | 2 | 3 | 4 | 5 |
| 27. When I see someone who badly needs help in an emergency, I go to pieces. | 1 | 2 | 3 | 4 | 5 |
| 28. Before criticizing somebody, I try to imagine how I would feel if I were in their place. | 1 | 2 | 3 | 4 | 5 |
|  |  |  |  |  |  |

The Davis Interpersonal Reactivity Index (IRI) used to assess empathic Concern and Perspective taking analysed in this paper. Davis, MH. (1983). Measuring individual differences in empathy: Evidence for a multidimensional approach*.* J Pers Soc Psychol, **44**, 113-126 (doi. [http://dx.doi.org/10.1037/0022-3514.44.1.113](http://psycnet.apa.org/doi/10.1037/0022-3514.44.1.113)).

**[2] Instructions and Materials for Study 2**

Perceptions of Males Responses to other’s Unfairness

Imagine this scenario involving 3 people (A, B & C). Person A has been given £10 and told that they can share some, none or all of it with person B. Person A decides to give person B £2.

1. **To what extent do you think Person A treated Person B unfairly**

**Not at all** 1 2 3 4 5 6 7 **Completely**

Person C has been given £5 and can choose to keep all £5 or spend some to punish Person A or compensate Person B. Every £1 Person C spends to punish person A will result in Person A losing £2 and every £1 Person C spends to compensate person B will result in person B gaining £2.

1. **Imagine person C is a male, if you were looking for a *short term* relationship to what extent would you find him more attractive if:**
2. He decided to spend his money punishing player A

**Not at all attractive** 1 2 3 4 5 6 7 **Very Attractive**

1. He decided to spend his money compensating player B

**Not at all attractive** 1 2 3 4 5 6 7 **Very Attractive**

1. He decided to spend his money doing a mixture of punishing player A and compensating player B

**Not at all attractive** 1 2 3 4 5 6 7 **Very Attractive**

1. He decided to keep the money for himself

**Not at all attractive** 1 2 3 4 5 6 7 **Very Attractive**

**3. Imagine person C is a male, if you were looking for a *long term* relationship would you find him more attractive if:**

1. He decided to spend his money punishing player A

**Not at all attractive** 1 2 3 4 5 6 7 **Very Attractive**

1. He decided to spend his money compensating player B

**Not at all attractive** 1 2 3 4 5 6 7 **Very Attractive**

1. He decided to spend his money doing a mixture of punishing player A and compensating player B

**Not at all attractive** 1 2 3 4 5 6 7 **Very Attractive**

1. He decided to keep the money for himself

**Not at all attractive** 1 2 3 4 5 6 7 **Very Attractive**

The following, are questions about your personal background. You should attempt to fill in all the answers. However, if you prefer not to, or cannot answer a particular question, please leave it blank.

Age: ______ Are you currently in a relationship: Yes No

What is your sexual orientation? a) Heterosexual, b) Homosexual, c) Bisexual (*Please circle as appropriate).*

**[3] Instructions and Materials for Study 3**

Perceptions of Males Responses to other’s Unfairness

Imagine this scenario involving 3 people (A, B & C). Person A has been give £10 and told that they can share some, none or all of it with person B. Person A decides to give person B £2.

1. **To what extent do you think Person A treated Person B unfairly**

**Not at all** 1 2 3 4 5 6 7 **Completely**

Person C has been given £5 and can choose to keep all £5 or spend some to punish Person A or compensate Person B. Every £1 Person C spends to punish person A will result in Person A losing £2 and every £1 Person C spends to compensate person B will result in person B gaining £2.

1. **Imagine person C is a male.**
2. Imagine **he** decided to spend his **money punishing player A**. If you were looking for a ***short term* relationship** to what extent would find **him**

| ***Unattractive*** |  |  | ***Neither Attractive nor Unattractive*** |  |  | ***Very Attractive*** |
| --- | --- | --- | --- | --- | --- | --- |
| 1 | 2 | 3 | 4 | 5 | 6 | 7 |
| ***Uncompassionate*** |  |  | ***Neither compassionate nor uncompassionate*** |  |  | ***Very Compassionate*** |
| 1 | 2 | 3 | 4 | 5 | 6 | 7 |
| ***Unfair*** |  |  | ***Neither fair nor unfair*** |  |  | ***Very Fair*** |
| 1 | 2 | 3 | 4 | 5 | 6 | 7 |
| ***Weak*** |  |  | ***Neither weak nor strong*** |  |  | ***Strong*** |
| 1 | 2 | 3 | 4 | 5 | 6 | 7 |

1. Imagine **he** decided to spend his money **compensating player B.** If you were looking for a ***short term* relationship** to what extent would find **him**

| ***Unattractive*** |  |  | ***Neither Attractive nor Unattractive*** |  |  | ***Very Attractive*** |
| --- | --- | --- | --- | --- | --- | --- |
| 1 | 2 | 3 | 4 | 5 | 6 | 7 |
| ***Uncompassionate*** |  |  | ***Neither compassionate nor uncompassionate*** |  |  | ***Very Compassionate*** |
| 1 | 2 | 3 | 4 | 5 | 6 | 7 |
| ***Unfair*** |  |  | ***Neither fair nor unfair*** |  |  | ***Very Fair*** |
| 1 | 2 | 3 | 4 | 5 | 6 | 7 |
| ***Weak*** |  |  | ***Neither weak nor strong*** |  |  | ***Strong*** |
| 1 | 2 | 3 | 4 | 5 | 6 | 7 |

1. Imagine **he** decided to spend his money doing a **mixture of punishing player A and compensating player B.** If you were looking for a ***short term* relationship** to what extent would find **him**

| ***Unattractive*** |  |  | ***Neither Attractive nor Unattractive*** |  |  | ***Very Attractive*** |
| --- | --- | --- | --- | --- | --- | --- |
| 1 | 2 | 3 | 4 | 5 | 6 | 7 |
| ***Uncompassionate*** |  |  | ***Neither compassionate nor uncompassionate*** |  |  | ***Very Compassionate*** |
| 1 | 2 | 3 | 4 | 5 | 6 | 7 |
| ***Unfair*** |  |  | ***Neither fair nor unfair*** |  |  | ***Very Fair*** |
| 1 | 2 | 3 | 4 | 5 | 6 | 7 |
| ***Weak*** |  |  | ***Neither weak nor strong*** |  |  | ***Strong*** |
| 1 | 2 | 3 | 4 | 5 | 6 | 7 |

1. Imagine **he** decided to **keep the money for himself**. If you were looking for a ***short term* relationship** to what extent would find **him**

| ***Unattractive*** |  |  | ***Neither Attractive nor Unattractive*** |  |  | ***Very Attractive*** |
| --- | --- | --- | --- | --- | --- | --- |
| 1 | 2 | 3 | 4 | 5 | 6 | 7 |
| ***Uncompassionate*** |  |  | ***Neither compassionate nor uncompassionate*** |  |  | ***Very Compassionate*** |
| 1 | 2 | 3 | 4 | 5 | 6 | 7 |
| ***Unfair*** |  |  | ***Neither fair nor unfair*** |  |  | ***Very Fair*** |
| 1 | 2 | 3 | 4 | 5 | 6 | 7 |
| ***Weak*** |  |  | ***Neither weak nor strong*** |  |  | ***Strong*** |
| 1 | 2 | 3 | 4 | 5 | 6 | 7 |

The following statements inquire about your thoughts and feelings in a variety of situations. For each item, indicate how well it describes you by circling the appropriate number on the scale. Answer as honestly as you can.

|  | **Does not describe me very well** | |  |  |  | **Describes me very well** | |
| --- | --- | --- | --- | --- | --- | --- | --- |
| I often have tender, concerned feelings for people less fortunate than me | 1 | 2 | 3 | 4 | 5 | 6 | 7 |
| When I see someone being taken advantage of, I feel kind of protective towards them | 1 | 2 | 3 | 4 | 5 | 6 | 7 |
| I am often quite touched by things I see happen | 1 | 2 | 3 | 4 | 5 | 6 | 7 |
| I would describe myself as a pretty soft-hearted person | 1 | 2 | 3 | 4 | 5 | 6 | 7 |

The following, are questions about your personal background. You should attempt to fill in all the answers. However, if you prefer not to, or cannot answer a particular question, please leave it blank.

*
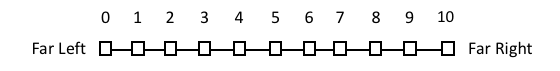
*In politics, people often talk about “left” and “right” when describing different political views. When you think about your own political views, how would you rate them on the scale below?

Age: ______

Are you currently in a relationship: Yes No

What is your sexual orientation? a) Heterosexual, b) Homosexual, c) Bisexual (*Please circle as appropriate).*

Perceptions of Males Responses to other’s Unfairness

Imagine this scenario involving 3 people (A, B & C). Person A has been give £10 and told that they can share some, none or all of it with person B. Person A decides to give person B £2.

1. **To what extent do you think Person A treated Person B unfairly**

**Not at all** 1 2 3 4 5 6 7 **Completely**

Person C has been given £5 and can choose to keep all £5 or spend some to punish Person A or compensate Person B. Every £1 Person C spends to punish person A will result in Person A losing £2 and every £1 Person C spends to compensate person B will result in person B gaining £2.

1. **Imagine person C is a male.**
2. Imagine **he** decided to spend his money **punishing player A**. If you were looking for a ***long term* relationship** to what extent would find **him**

| ***Unattractive*** |  |  | ***Neither Attractive nor Unattractive*** |  |  | ***Very Attractive*** |
| --- | --- | --- | --- | --- | --- | --- |
| 1 | 2 | 3 | 4 | 5 | 6 | 7 |
| ***Uncompassionate*** |  |  | ***Neither compassionate nor uncompassionate*** |  |  | ***Very Compassionate*** |
| 1 | 2 | 3 | 4 | 5 | 6 | 7 |
| ***Unfair*** |  |  | ***Neither fair nor unfair*** |  |  | ***Very Fair*** |
| 1 | 2 | 3 | 4 | 5 | 6 | 7 |
| ***Weak*** |  |  | ***Neither weak nor strong*** |  |  | ***Strong*** |
| 1 | 2 | 3 | 4 | 5 | 6 | 7 |

1. Imagine **he** decided to spend his money **compensating player B**. If you were looking for a ***long term* relationship** to what extent would find **him**

| ***Unattractive*** |  |  | ***Neither Attractive nor Unattractive*** |  |  | ***Very Attractive*** |
| --- | --- | --- | --- | --- | --- | --- |
| 1 | 2 | 3 | 4 | 5 | 6 | 7 |
| ***Uncompassionate*** |  |  | ***Neither compassionate nor uncompassionate*** |  |  | ***Very Compassionate*** |
| 1 | 2 | 3 | 4 | 5 | 6 | 7 |
| ***Unfair*** |  |  | ***Neither fair nor unfair*** |  |  | ***Very Fair*** |
| 1 | 2 | 3 | 4 | 5 | 6 | 7 |
| ***Weak*** |  |  | ***Neither weak nor strong*** |  |  | ***Strong*** |
| 1 | 2 | 3 | 4 | 5 | 6 | 7 |

1. Imagine **he** decided to spend his money doing a **mixture of punishing player A and compensating player B**. If you were looking for a ***long term* relationship** to what extent would find **him**

| ***Unattractive*** |  |  | ***Neither Attractive nor Unattractive*** |  |  | ***Very Attractive*** |
| --- | --- | --- | --- | --- | --- | --- |
| 1 | 2 | 3 | 4 | 5 | 6 | 7 |
| ***Uncompassionate*** |  |  | ***Neither compassionate nor uncompassionate*** |  |  | ***Very Compassionate*** |
| 1 | 2 | 3 | 4 | 5 | 6 | 7 |
| ***Unfair*** |  |  | ***Neither fair nor unfair*** |  |  | ***Very Fair*** |
| 1 | 2 | 3 | 4 | 5 | 6 | 7 |
| ***Weak*** |  |  | ***Neither weak nor strong*** |  |  | ***Strong*** |
| 1 | 2 | 3 | 4 | 5 | 6 | 7 |

1. Imagine **he** decided to **keep the money for himself**. If you were looking for a ***long term* relationship** to what extent would find **him**

| ***Unattractive*** |  |  | ***Neither Attractive nor Unattractive*** |  |  | ***Very Attractive*** |
| --- | --- | --- | --- | --- | --- | --- |
| 1 | 2 | 3 | 4 | 5 | 6 | 7 |
| ***Uncompassionate*** |  |  | ***Neither compassionate nor uncompassionate*** |  |  | ***Very Compassionate*** |
| 1 | 2 | 3 | 4 | 5 | 6 | 7 |
| ***Unfair*** |  |  | ***Neither fair nor unfair*** |  |  | ***Very Fair*** |
| 1 | 2 | 3 | 4 | 5 | 6 | 7 |
| ***Weak*** |  |  | ***Neither weak nor strong*** |  |  | ***Strong*** |
| 1 | 2 | 3 | 4 | 5 | 6 | 7 |

The following statements inquire about your thoughts and feelings in a variety of situations. For each item, indicate how well it describes you by circling the appropriate number on the scale. Answer as honestly as you can.

|  | **Does not describe me very well** | |  |  |  | **Describes me very well** | |
| --- | --- | --- | --- | --- | --- | --- | --- |
| I often have tender, concerned feelings for people less fortunate than me | 1 | 2 | 3 | 4 | 5 | 6 | 7 |
| When I see someone being taken advantage of, I feel kind of protective towards them | 1 | 2 | 3 | 4 | 5 | 6 | 7 |
| I am often quite touched by things I see happen | 1 | 2 | 3 | 4 | 5 | 6 | 7 |
| I would describe myself as a pretty soft-hearted person | 1 | 2 | 3 | 4 | 5 | 6 | 7 |

The following, are questions about your personal background. You should attempt to fill in all the answers. However, if you prefer not to, or cannot answer a particular question, please leave it blank.

*
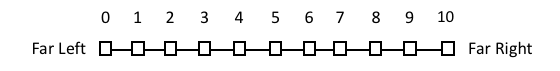
*In politics, people often talk about “left” and “right” when describing different political views. When you think about your own political views, how would you rate them on the scale below?

Age: ______

Are you currently in a relationship: Yes No

What is your sexual orientation? a) Heterosexual, b) Homosexual, c) Bisexual (*Please circle as appropriate).*

**[4]Cultural context of the Studies**: **Nottingham University and Nottingham**

Our samples are convenience samples taken from students at Nottingham University (<http://www.nottingham.ac.uk/>) which is one of the 24 UK Russell Group Universities (<http://russellgroup.ac.uk/>). Nottingham is situated in the East Midlands. It is a UNESCO city of literature (https://nottinghamcityofliterature.com/). Employment statistics of Nottingham can be found at <https://www.nomisweb.co.uk/reports/lmp/la/1946157131/printable.aspx>. The ethnic mix and other demographic information for Nottingham University can be found at <http://www.nottingham.ac.uk/spp/student-statistics/detailed-statistics-and-analyses-201617-pdf.aspx>.

**Section B: Equivalence of Pure Control and Active Control for Priming**

**Results**: The results, comparing the ‘pure-control’, ‘romantic-prime’ and ‘active control’ (street scene) are shown in Figure S1 and Table S1. These provide the percentage of participants choosing each preference.

***Pure Control vs Active Control ‘***: As can be seen the pattern of results observed comparing the ‘pure control’ and the ‘active control’ (street scenes) are virtually identical and not significantly different from each other.

***Romantic Prime vs Active Control***: The results for the ‘romantic prime’ vs the ‘active control’ (street scene) shows that men exposed to the ‘active control’ got priming are more likely to express a preference to ‘do-nothing’ or to ‘punish’, where s men exposed to the romantic prime are more likely to show a preference to ‘compensate’.

***Romantic Prime vs Pure Control***: The results for the ‘romantic prime’ vs the ‘pure control’ show that men exposed to the ‘pure control’ are more likely to express a preference to ‘do-nothing’ or top ‘punish’, where s men exposed to the ‘romantic prime’ are more likely to show a preference to ‘compensate’ or both ‘compensate and punish’.

Given that the ‘pure control’ and the ‘active control’ did not differ, we combined these data into a single ‘combined control’ and compared the pattern to that observed in the ‘romantic prime’.

**Legend**: Figures in parenthesis are the number of participants choosing each preference

**Table S1:** Yates Corrected χ^2^ Exploring the Pairwise Differences between the prime-control, the pure-control and romantic-prime

| **Preference** | **Pure-Control vs Active Control-Prime** | φ | **Active Control Prime Vs Romantic Prime** | φ | **Pure Control Vs Romantic Prime** | φ |
| --- | --- | --- | --- | --- | --- | --- |
| Do Nothing | 0.038 (p = .846) | .065 | 8.080 (p = .004) | .436 | 11.82 (p = .000) | .48 |
| Punish | 0.000 (p = 1.0) | .005 | 5.495 (p = .019) | .375 | 5.88 (p = .015) | .36 |
| Compensate | 0.000 (p = 1.0) | .004 | 8.769 (p = .003) | .446 | 10.55 (p = .001) | .45 |
| Both Punish and Compensate | 0.098 (p = .754) | .108 | 1.903 (p = .168) | .234 | 5.10 (p = .024) | .33 |

**Section C: Interpretation of Attractiveness Ratings for Study Two**

The attractiveness rating were made in a 7 point scale (1 = not attractive to 7 = very attractive). Thus ratings below 3.5 could indicate judgments of more or less unattractiveness and above as more of less attractive. To explore this we ran a series of one-sample T-test with 3.5 as the reference value (see Table S2).

**Table S2**. *One Sample T-test (reference value = 3.5) for attractiveness ratings*

|  | Mean | t_(one sample)_ 3.5 | p = | Cohen’s d |
| --- | --- | --- | --- | --- |
| **Short-term** |  |  |  |  |
| Punish | 2.73 | -4.98 | .000 | 0.49 |
| Compensate | 5.52 | 18.19 | .000 | 1.94 |
| Compensate & Punish | 4.00 | 2.99 | .003 | 0.29 |
| Do Nothing | 2.65 | -5.52 | .000 | 0.54 |
| **Long-term** |  |  |  |  |
| Punish | 2.26 | -9.22 | .000 | 0.90 |
| Compensate | 5.65 | 16.73 | .000 | 1.64 |
| Compensate & Punish | 3.90 | 2.25 | .027 | 0.22 |
| Do Nothing | 2.49 | -6.43 | .000 | 0.62 |

As can be seen in Table S2 ‘punishment’ and ‘do Nothing’, for both long-term and short-term relationships, are rated significant lower than 3.5 and maybe thus interpreted as indicating more or less unattractiveness. ‘Compensate’ and ‘Compensate & Punish’, for both long-term and short-term relationships, are rated greater than 3.5 and may be interpreted as indicating attractive.

**Section D: Additional Analyses for Studies One, Two and Three**

**[1]. Additional Analysis for Study One**

**Spend on Compensation and Punishment within the ‘both compensate and punish preference’ in Study One**

Table S3 below shows, for those who chose the ‘both compensate and punish’ preference, the amount they decided to spend on punishment and compensation. In the romantic-prime, the dominant distribution was £1 for punishment and £1 for ‘compensation’ or to give slightly more to compensation (£2) than punishment (£1). In the pure-control the only distribution was £1 for punishment and £1 for compensation. In the control-prime it was either £1 for punishment and £1 for compensation or a slight preference to punish (£3) rather than compensate (£2).

**Table S3.** Frequency of spending choices in the ‘both compensate and punish’ preference by condition.

|  | Compensate and punish | | |
| --- | --- | --- | --- |
|  | Pure-Control | Romantic-Prime | Active-Control Prime |
| N choosing: |  |  |  |
| £1 Compensation & £1 punishment | 2 | 6 | 2 |
| £2 Compensation & £1 punishment | 0 | 3 |  |
| £1 Compensation & £2 punishment | 0 | 1 |  |
| £2 Compensation & £3 punishment | 0 | 0 | 1 |
| Average spend on compensation | £1 | £1.3 | £1.3 |
| Average spend on punishment | £1 | £1.1 | £1.6 |
| Total N | 2 | 10 | 3 |

**Effect of Relationship Status and Comprehension Tests in Study One**

In Study One 5 of the 60 original participants required help to understand the 3PPC game (2 in the prime and 3 in the pure-control). In the additional active control 6 needed help. Twenty-one single males were in the pure-control and 20 in the romantic-prime condition (χ^2^ = (1) 0.08, p = 1.0: φ= .04). There were 18 single males in the additional ‘activecontrol’. Participants in the romantic-prime condition took on average 2 minutes and 37 seconds (SD = 0.30 seconds) to complete the prime, with 53% taking all 3 minutes. The 23 participants in the control-prime took on average 2 minutes and 31 seconds (SD = 0.36 seconds) to complete the prime, with 52% taking all 3 minutes. Time to complete the ‘romantic-prime’ did not differ significantly from the time taken to complete the ‘active control’ prime (*t* _(51)_ = 0.71, p = .480: Cohen’s d = 0.182).

**Relationship Status: being Single**

Table S4, below, shows that whether or not the men in study one were single or not, did not influence preference choice overall or within each prime condition.

**Table S4**: *Effects of Relationship Status (Single or not) on Preferences in Study 1*

|  | Yates Corrected χ^2^ | Fisher’s Exact p | φ |
| --- | --- | --- | --- |
| **Do Nothing** |  |  |  |
| Romantic Prime * Single |  | .083 | .408 |
| Pure Control * Single | 0.000 (p = 1.0) | 1.0 | .000 |
| Active Control * Single |  | .339 | .250 |
| Total effect of being single on Preference |  | 1.0 | .011 |
| **Punish** |  |  |  |
| Romantic Prime * Single |  | 1.00 | .122 |
| Pure Control * Single |  | .431 | .154 |
| Active Control * Single |  | .621 | .110 |
| Total effect of being single on Preference |  | .555 | .07 |
| **Compensate** |  |  |  |
| Romantic Prime * Single |  | 1.0 | .015 |
| Pure Control * Single |  | 1.0 | .069 |
| Active Control * Single |  | .536 | .109 |
| Total effect of being single on Preference |  | 1.0 | .004 |
| **Both Punish & Compensate** |  |  |  |
| Romantic Prime * Single |  | .675 | .154 |
| Pure Control * Single |  | .540 | .189 |
| Active Control * Single |  | .539 | .109 |
| Total effect of being single on Preference |  | .536 | .092 |

***Note***. N for Romantic Prime * Single = 30; Romantic Prime * Single = 30; Active Control * Single = 23and Total effect = 83

**Analysis of Free-Response Data form the Romantic Primes in Study One**

The primed participant’s narratives about their ideal date, were explored to identify the major themes and the following coding frame developed.

1. ***Self vs other orientation*** (0 = self, 1 = other). Focus on what the writer wants (e.g., I *want to do X because I enjoy it, I like, personality I would*, I think X is the best ever etc.) rather than the women (date; e.g., they might like, they want to do etc.)
2. ***Other-Oriented: Focus on trying to find out more about the date*** ***(woman) (****conversation, chat, get to know each other, talk etc.****)*** (0 = no , 1 = yes)
3. ***Focus on the context (where, what to eat etc.)*** (0 = no , 1 = yes)
4. ***Mention having a nice time*** (0 = no, 1 = yes)
5. ***Mentions positive emotions/mood (e.g., happy, laughter, enjoyment)*** (0 = no, 1 = yes)
6. ***Mentions would like a 2^nd^date.*** (0 = no 1 = yes)
7. ***Setting a positive mood (e.g., bring flowers, initial icebreaker activity)*** (0 = no, 1 = yes)
8. ***Costly signal/status (e.g., I would pay, expensive place, expensive experience, try to impress) vs not (e.g., standard bar etc.)*** (0 = no, 1 = yes)
9. ***Try to create romantic intimate atmosphere*** (0 = no, 1 = yes)
10. ***How nice person:*** *1 = not very to 10 very*
11. ***How nice the date:*** *1 = not very to 10 very*

This frame was applied to all 30 narratives by two female raters (FH the 2^nd^ author) and a Senior Academic working on sexual selection, aggression and mate choice. Both were blind to the choices made by each participant. Disagreements were resolved by the 1^st^ author (also blind to choices made by the participants). On average narrative were 38.9 words long (SD = 18.79). The results are show in the Table S5 below.

Based on Landis and Koch’c (1977) interpretative scheme for categorical inter-rater reliability all kappa coefficients are substantial to almost perfect apart from: (1) Mention having a nice time, (2) Setting a positive mood and (3) Try to create romantic intimate atmosphere. However, these all had a low endorsement rate as well.

Reliably 50% of narratives were *other oriented*, focusing on trying to get to know more about the women and 27% displaying a costly signal or status. The participants were rated on average as nice (5.6 to 6.1).

**Table S5***: Narrative themes and reliability*

| ***Theme*** | ***%, Mean (SD)*** | ***Reliability*** |
| --- | --- | --- |
| ***Self vs other orientation*** (0 = self, 1 = other). | *13%* | *100%* |
| ***Other-Oriented (***0 = no, 1 = yes) | *50%* | *k = .93* |
| ***Focus on the context (where, what to eat etc.)*** | *100%* | *97%* |
| ***Mention having a nice time*** (0 = no, 1 = yes) | *6%* | *k = .35* |
| ***Mentions positive emotions/mood (e.g., happy, laughter, enjoyment)*** (0 = no, 1 = yes) | *17%* | *k = .67* |
| ***Mentions would like a 2^nd^date.*** (0 = no 1 = yes) | *13%* | *100%* |
| ***Setting a positive mood (e.g., bring flowers, initial icebreaker activity)*** (0 = no, 1 = yes) | *10%* | *k = .47%* |
| ***Costly signal/status (e.g., I would pay, expensive place, expensive experience, try to impress) vs not (e.g., standard bar etc.)*** (0 = no, 1 = yes) | *27%* | *k = .73%* |
| ***Try to create romantic intimate atmosphere*** (0 = no, 1 = yes) | *20%* | *0%* |
| ***How nice person:*** *1 = not very to 10 very* | *6.1(0.71) rater 1*  *5.6 (1.54) rater 2* | *r = .73* |
| ***How nice the date:*** *1 = not very to 10 very* | *5.9 (0.97) rater 1*  *6.0 (2.32) rater 2* | *r = .49* |

*k= Kappa Reliability*

Interpretation of kappa coefficient from From Landis and Koch (1977)

***Kappa Statistic Strength of Agreement***

< 0.00 Poor

0.00-0.20 Slight

0.21-0.40 Fair

0.41-0.60 Moderate

0.61-0.80 Substantial

0.81-1.00 Almost Perfect

**References**

Griskevicius V, Tybur JM, Sundie JM, Cialdini RB, Miller GF, Kenrick DT. 2007. Blatant benevolence and conspicuous consumption: When romantic motives elicit strategic costly signals. *J. Pers. Soc. Psychol*. **93**, 85-102. (doi:10.1037/0022-3514.93.1.85)

Landis JR. Gary G. Koch, GG. (1977). The Measurement of Observer Agreement for Categorical Data. Biometrics. **33**, 159-174 (doi. <http://www.jstor.org/stable/2529310>).

**Association Between Traits and Emotions in Study One.**

Table S6 shows the associations between trait and emotional assessments in Study One.

**Table S6***: Associations (Spearman’s) between the trait and Emotion Measures in Study One*

|  | (1) | (2) | (3) | (4) | (5) | (6) |
| --- | --- | --- | --- | --- | --- | --- |
| ***Traits*** |  |  |  |  |  |  |
| Empathic Anger (1) | 1 |  |  |  |  |  |
| Empathic Concern (2) | .39** | 1 |  |  |  |  |
| Perspective Taking (3) | .17 | .37** | 1 |  |  |  |
| ***Emotions*** |  |  |  |  |  |  |
| Moral outrage (4) | .24* | .21 | .07 | 1 |  |  |
| Empathic Concern (5) | .18 | .47*** | .28** | .38** | 1 |  |
| Empathic Distress (6) | .31** | .35** | .13 | 69*** | .49*** | 1 |
| Empathic Sadness (7) | .25* | .35** | .09 | .57*** | .56*** | .69*** |

***Note***. * p < .05, **, P , .01, *** P < .001

As can be seen trait empathic concern was associated with trait empathic anger and perspective taking. Moral outrage wass associated with trait empathic anger. All the emotional assessment that focus on reaction to Player A’s unfairness are positively associated with each other. Moral outrage is linked not only to more negative emotions (sadness and distress) but also more compassionate emotion’s (empathic concern).

**[2]. Additional Analysis for Study Two**

***Effects of relationship type, preference and their interaction reported in the main paper are altered when relationship status (singe or not) and the study setting (individual or group***

These initial analyses explore if the main effects of *relationship type, preference* and their interaction reported in the main paper are altered when relationship *status* (singe or not) and the study *setting* (individual or group). Thus we conducted a 2 (*relationship type*: short-term vs long-term) by 4 (*preference*: punish, compensate, both or nothing) by *status* (singe or not) by *setting* (individual or group) mixed effects ANOVA. Where *relationship type* and *preference* were within subjects factors and *status* and *setting* between subjects factors. The results are shown in Table 7. As can be seen the inclusion of the two between subjects factors, *status* (single or not) and *setting* (individual or group), did not alter any of the main or interactive effects of *relationship type* or *preference* reported in the analyses in the main paper. There is an additional between subjects main effect for *setting*, such that those who completed the task in a group setting had a tendency overall to report higher attractiveness levels (*M* = 3.943, *Se* = 0.124, *95%CIs* = 3.696, 4.190) than those tested individually *M* = 3.451, *Se* = 0.110, *95%CIs* = 3.232, 3.670).

**Table S**7 *Mixed Within-Between ANOVA for Attractiveness Ratings with Setting and Status as Additional Between Subjects Factors*

|  | df | F | p | ε^2^p |
| --- | --- | --- | --- | --- |
| **Within Subjects** |  |  |  |  |
| ***Relationship type*** | **1** | **7.505** | **.007** | **.070** |
| *Relationship type * Status* | 1 | 0.000 | .984 | .000 |
| *Relationship type * Setting* | 1 | 0.006 | .938 | .000 |
| *Relationship type * Status * Setting* | 1 | 0.021 | .885 | .000 |
| Error (*Relationship type)* | 99 |  |  |  |
| ***Preference*** | **3** | **113.511** | **.000** | **.534** |
| *Preference * Status* | 3 | 1.607 | .188 | .016 |
| *Preference * Setting* | 3 | 2.056 | .106 | .020 |
| *Preference * Status * Setting* | 3 | 0.484 | .694 | .005 |
| *Preference* (*Relationship type)* | 297 |  |  |  |
| ***Relationship type * Preference*** | **3** | **5.514** | **.001** | **.053** |
| *Relationship type * Preference * Status* | 3 | 0.318 | .813 | .003 |
| *Relationship type * Preference * Setting* | 3 | 0.781 | .505 | .008 |
| *Relationship type * Preference * Status * Setting* | 3 | 1.125 | .339 | .011 |
| Error (*Relationship type* Preference)* | 297 |  |  |  |
| *Between Subjects Effects* |  |  |  |  |
| *Status* | 1 | 1.303 | .256 | .013 |
| ***Setting*** | **1** | **8.746** | **.004** | **.081** |
| *Status * Setting* | 1 | 0.002 | .962 | .000 |
| Error | 99 |  |  |  |

**[3]. Additional Analysis for Study Three**

Sensitive and Robustness Checks for Interaction with Empathic Concern and Political Ideology.

**Dichotomizing empathic concern and political ideology**: Those who scored 22 or less on empathic concern were classed a lower on empathic concern (N = 75) and those who scores 23 or more as higher (N = 85) with one missing value. Those who scored 3 or less on the political ideology scale were classed more left-wing (N = 65) and those who scored 4 or more as more right-wing (N = 80) with 16 missing values. We entered these into a full factorial MANOVA with relationship-type and preference.

**MANOVA for Empathic Concern**: For *empathic concern* we ran a 2 between (*relationship type:* short-term vs long-term) by 4 within (*preference:* punish, compensate, both or nothing) by 2 between (*empathic concern*: high vs low) multivariate mixed MANOVA on ratings of attractiveness, compassion, fairness and strength (N = 158). Mauchly’s test of Sphericity were significant for *preference* for each of the ratings (attraction, compassion, fairness and strength: all *p*’s = .000 and all Epsilon’s great then .75). As Sphericity cannot be assured Epsilon adjusted effects are reported for within subject effects. There was a significant main effect for *preference* (*F _Pillai’s Trace_* _(12, 1383)_ = 32.81, *p* = .000, ε^2^p = .222), but no significant interaction *between preference* and *relationship* *type* (*F _Pillai’s Trace_* _(12, 1383)_ = 1.65, *p* = .073, ε^2^p = .014). The significant preference effect was observed for all 4 ratings: (1) attractiveness (*F _Huynh-Feldt_* _(2.67, 411.03)_ = 129.63, *p* = .000, ε^2^p = .457), (2) compassion (*F _Huynh-Feldt_* _(2.69, 413.99)_ = 217.81, *p* = .000, ε^2^p = .586), (3) fairness (*F _Huynh-Feldt_* _(2.77, 427.69)_ = 95.84, *p* = .000, ε^2^p = .384), and (4) strength (*F _Huynh-Feldt_* _(2.82, 435.57)_ = 39.53, *p* = .000, ε^2^p = .204) and is the same as in Figure 3.

There was significant main effects for *relationship* type (*F _Pillai’s Trace_* _(4, 151)_ = 4.38, *p* = .002, ε^2^p = .104), with a significant univariate effect for attractiveness (*F* _(31, 154)_ = 4.19, *p* = .042, ε^2^p = .026) which was preferred for a short-term relationship (3.72 _short-term_ [95%CI 3.57, 3.87] vs 3.50 _long-term_ [95%CI 3.35, 3.65]).

In addition there was an interaction between *empathic concern* and *preference* (*F _Pillai’s Trace_* _(12, 1383)_ = 4.23, *p* = .001, ε^2^p = .04). The interaction was significant for all 4 rating: (1) attraction (*F _Huynh-Feldt_* _(2.67, 411.30)_ = 8.29, *p* = .000, ε^2^p = .051), (2) compassion (*F _Huynh-Feldt_* _(2.69, 413.99)_ = 6.83 *p* = .000, ε^2^p = .042), (3) fairness (*F _Huynh-Feldt_* _(2.78, 427.69)_ = 7.27, *p* = .000, ε^2^p = .045) and (4) strength (*F _Huynh-Feldt_* _(2.83,4 35.567)_ = 12.60, *p* = .000, ε^2^p = .076). This interaction (Figure 4) shows that those higher in trait empathic concern there are significantly more likely to rate male who do nothing as more ‘unattractive’, ‘uncompassionate’, ‘unfair’ or ‘weak’. Those who score high on empathic concern are also more likely to perceived males who punish as more unattractive. Finally those higher in empathic concerns view male who compensate as strong. Thus, the pattern of findings is the same when empathic concern is included as a factor and shows that additionally empathic concern adds to the prediction on preferred distributive justice strategy.

**Figure S2.** *Interaction of Relationship Tyupe by Preference by Empathic Concern*

**MANOVA for Empathic Concern**: For *political ideology* we ran a 2 between (*relationship type:* short-term vs long-term) by 4 within (*preference:* punish, compensate, both or nothing) by 2 between (*political ideology*: left vs right wing) subjects multivariate mixed MANOVA on all 4 ratings (N = 144). Mauchly’s test of Sphericity were significant for *preference* for each of the ratings (attraction, compassion, fairness and strength: all *p*’s = .000 and all Epsilon’s great then .75). As Sphericity cannot be assured Epsilon adjusted effects are reported for within subject effects. There was a significant main effects for *preference* (*F _Pillai’s Trace_* _(12, 1257)_ = 30.45, *p* = .000, ε^2^p = .225) and no significant interaction *between preference* and *relationship* type (*F _Pillai’s Trace_* _(12, 1257)_ = 1.69, *p* = .062, ε^2^p = .016). The effect of preference effect was again significant for all 4 ratings: (1) attractiveness (*F _Huynh-Feldt_* _(2.71, 379.00)_ = 122.78, *p* = .000, ε^2^p = .467), (2) compassion (*F _Huynh-Feldt_* _(2.75, 384.90)_ = 212.62, *p* = .000, ε^2^p = .803), (3) fairness (*F _Huynh-Feldt_* _(2.77, 388.20)_  = 92.47, *p* = .000, ε^2^p = .398), and (4) strength (*F _Huynh-Feldt_* _(2.77, 387.83)_ = 42.44, *p* = .000, ε^2^p = .233), with the same pattern as in Figure 3..

There was significant main effect for *relationship* type (*F _Pillai’s Trace_* _(4, 137)_ = 3.89, *p* = .005, ε^2^p = .102), In terms of relationship type at the univariate level there were no significant effects.

In addition there was an interaction between *political ideology* and *preference* (*F _Pillai’s Trace_* _(12, 1257)_ = 1.81, *p* = .042, ε^2^p = .017). The interaction was significant for rating of attraction (*F _Huynh-Feldt_* _(2.70, 379.00)_ = 3.39, *p* = .022, ε^2^p = .024) and strength (*F _Huynh-Feldt_* _(2.77, 387.83)_ = 4.22, *p* = .007, ε^2^p = .029). This interaction is shown in Figure 5. Women with a left-wing ideology were more likely to find men attractive if they chose to ‘compensate’ and those with a right-wing ideology more likely to find men who chose to ‘do-nothing’ as strong.

**Figure S3.** *Interaction of Relationship Tyupe by Preference by Political Ideology*

**Effects of Relationship Status**

Adding relationship status to this model and running a fully factorial model indicates that relationship status did not have a significant main effect on any of the ratings (*p*s range .992, .566), did not interact with preference (*F _Pillai’s Trace_* _(12, 1392)_ = 0.852, *p* = .0577, ε^2^p = .007) or relationship type (*F _Pillai’s Trace_* _(12, 1392)_ = 1.575, *p* = .092, ε^2^p = .013), nor was there as 3-wat interaction with relationship type and preference (*F _Pillai’s Trace_* _(12, 1392)_ = 1.611, *p* = .082, ε^2^p = .014).

**Section E: Details for GEE Models**

The modelling specifications for the Generalized Estimating Equations (*GEE*) models detailed in Section 3.3.5 are provided here.

**Distribution and Link Function**: The probability distribution was specified as normal with an identity link function.

**Model Parameters:** The target models contained main effects for *relationship type* (short-term vs log-term), *rating type* (attractive, compassionate, fair and strong), *preference* (‘punish’, ‘compensate’, ‘both compensate and punish’ and ‘do-nothing’), *Empathic Concern* or *Political Ideology*, and 2-way interactions between (1) *Relationship Type* and *Preference*, (2) *Rating Type* and *Preference* and (3) *Preference* and *Empathic Concern/political ideology*.

**Additional Parameters:** Additional 2-way and higher order interactions were also examined to explore if they provided additional explanatory power. This was assessed in terms of a reduction in information criteria (QIC) and individual parameter significance.

**Section F: Syntax for analyses linked to file Doi:** <https://datadryad.org/review?doi=doi:10.5061/dryad.738pm17>

Detailed below are the SPSS and M*Plus* syntax files for the data analysed in the main text and deposited at <https://datadryad.org/review?doi=doi:10.5061/dryad.738pm17>

**[1]. Study One**

***3.1.1 Pattern of Preference as a Function of Primes***

CROSSTABS

/TABLES=DV_Preference_Do_nothing DV_Preference_Punish DV_Preference_Compensate DV_Preference_Both

DV_Pref_Compensate_or_Both BY Experimental_conditions_combined_control_vs_romatic_prime

/FORMAT=AVALUE TABLES

/STATISTICS=CHISQ PHI

/CELLS=COUNT EXPECTED SRESID

/COUNT ROUND CELL.

**Multi-nominal models**

**Table 1**. *Preferences as a function Priming Condition, Affect, Traits and Mood*. Coefficients are unstandardized; Condition = combined control (0) vs romantic prime (1); reference group = do-nothing

**Model 1**

NOMREG DV_ordinal (BASE=FIRST ORDER=ASCENDING) BY Expt_condition_control_combined_reversed

/CRITERIA CIN(95) DELTA(0) MXITER(100) MXSTEP(5) CHKSEP(20) LCONVERGE(0) PCONVERGE(0.000001)

SINGULAR(0.00000001)

/MODEL=Expt_condition_control_combined_reversed

/STEPWISE=PIN(.05) POUT(0.1) MINEFFECT(0) RULE(SINGLE) ENTRYMETHOD(LR) REMOVALMETHOD(LR)

/INTERCEPT=INCLUDE

/PRINT=FIT PARAMETER SUMMARY LRT CPS STEP MFI.

**Model 2**

NOMREG DV_ordinal (BASE=FIRST ORDER=ASCENDING) BY Expt_condition_control_combined_reversed WITH

IRI_EMP_EC_total IRI_EMP_PT_total Empathic_anger_trait

/CRITERIA CIN(95) DELTA(0) MXITER(100) MXSTEP(5) CHKSEP(20) LCONVERGE(0) PCONVERGE(0.000001)

SINGULAR(0.00000001)

/MODEL=Expt_condition_control_combined_reversed IRI_EMP_EC_total IRI_EMP_PT_total

/STEPWISE=PIN(.05) POUT(0.1) MINEFFECT(0) RULE(SINGLE) ENTRYMETHOD(LR) REMOVALMETHOD(LR)

/INTERCEPT=INCLUDE

/PRINT=FIT PARAMETER SUMMARY LRT CPS STEP MFI.

**Model 3**

NOMREG DV_ordinal (BASE=FIRST ORDER=ASCENDING) BY Expt_condition_control_combined_reversed WITH

Moral_outrage_PA Empathic_concern_PA Empathic_distress_PA Empathic_sadness_PA

/CRITERIA CIN(95) DELTA(0) MXITER(100) MXSTEP(5) CHKSEP(20) LCONVERGE(0) PCONVERGE(0.000001)

SINGULAR(0.00000001)

/MODEL=Expt_condition_control_combined_reversed

/STEPWISE=PIN(.05) POUT(0.1) MINEFFECT(0) RULE(SINGLE) ENTRYMETHOD(LR) REMOVALMETHOD(LR)

/INTERCEPT=INCLUDE

/PRINT=FIT PARAMETER SUMMARY LRT CPS STEP MFI.

**Model 4**

NOMREG DV_ordinal (BASE=FIRST ORDER=ASCENDING) BY Expt_condition_control_combined_reversed WITH

IRI_EMP_EC_total IRI_EMP_PT_total Empathic_anger_trait Moral_outrage_PA Empathic_concern_PA

Empathic_distress_PA Empathic_sadness_PA

/CRITERIA CIN(95) DELTA(0) MXITER(100) MXSTEP(5) CHKSEP(20) LCONVERGE(0) PCONVERGE(0.000001)

SINGULAR(0.00000001)

/MODEL=Expt_condition_control_combined_reversed IRI_EMP_EC_total IRI_EMP_PT_total

Empathic_anger_trait Moral_outrage_PA Empathic_concern_PA Empathic_distress_PA Empathic_sadness_PA

/STEPWISE=PIN(.05) POUT(0.1) MINEFFECT(0) RULE(SINGLE) ENTRYMETHOD(LR) REMOVALMETHOD(LR)

/INTERCEPT=INCLUDE

/PRINT=FIT PARAMETER SUMMARY LRT CPS STEP MFI.

**[2]. Study Two**

**Sample Selection**

COMPUTE filter_$=(SexOrientation = 1).

VARIABLE LABELS filter_$ 'SexOrientation = 1 (FILTER)'.

VALUE LABELS filter_$ 0 'Not Selected' 1 'Selected'.

FORMATS filter_$ (f1.0).

FILTER BY filter_$.

EXECUTE.

***3.2.1. Perceived Fairness of the Transgression***

FREQUENCIES VARIABLES=unfairly

/STATISTICS=STDDEV MEAN

/ORDER=ANALYSIS.

T-TEST

/TESTVAL=1

/MISSING=ANALYSIS

/VARIABLES=unfairly

/CRITERIA=CI(.95).

***3.2.2. Attractiveness as a Function of Preference and Relationship Length***

GLM ST_punish ST_compensate ST_both ST_nothing LT_punish LT_compensate LT_both LT_nothing

/WSFACTOR=Type 2 Polynomial Preference 4 Polynomial

/METHOD=SSTYPE(3)

/EMMEANS=TABLES(OVERALL)

/EMMEANS=TABLES(Type)

/EMMEANS=TABLES(Preference)

/EMMEANS=TABLES(Type*Preference)

/PRINT=DESCRIPTIVE ETASQ OPOWER HOMOGENEITY

/CRITERIA=ALPHA(.05)

/WSDESIGN=Type Preference Type*Preference.

**[3]. Study 3**

**Sample selection**

USE ALL.

COMPUTE filter_$=(sexuality = 0).

VARIABLE LABELS filter_$ 'sexuality = 0 (FILTER)'.

VALUE LABELS filter_$ 0 'Not Selected' 1 'Selected'.

FORMATS filter_$ (f1.0).

FILTER BY filter_$.

EXECUTE.

***3.3.1 Perceived Fairness of the Transgression***

T-TEST

/TESTVAL=1

/MISSING=ANALYSIS

/VARIABLES=fairnerss_of_A

/CRITERIA=CI(.95).

***3.3.2*. *Fairness, Empathic Concern and Political Ideology***

NONPAR CORR

/VARIABLES=EC_total Political_ideology fairnerss_of_A

/PRINT=SPEARMAN TWOTAIL NOSIG

/MISSING=PAIRWISE.

***3.3.3. Ratings as a Function of Preference and Relationship Length***

GLM Punish_attractive Compen_attractive Mixed_attarctive Keep_attractive Punish_compass

Compen_compass Mixed_cmpass Keep_compass Punish_fair Compen_fair Mixed_fair Keep_fair Punish_strong

Compen_strong Mixed_strong Keep_strong BY Condition

/WSFACTOR=pref 4 Polynomial

/MEASURE=attrac comp fair strong

/METHOD=SSTYPE(3)

/EMMEANS=TABLES(OVERALL)

/EMMEANS=TABLES(Condition)

/EMMEANS=TABLES(pref)

/EMMEANS=TABLES(Condition*pref)

/PRINT=DESCRIPTIVE ETASQ OPOWER HOMOGENEITY

/CRITERIA=ALPHA(.05)

/WSDESIGN=pref

/DESIGN=Condition.

***3.3.5. Effects for Empathic Concern and Political Ideology***

***GEE analysis for Empathy***

GENLIN trans1 BY Condition rating_type Preference (ORDER=ASCENDING) WITH EC_total

/MODEL Condition rating_type Preference rating_type*Preference Condition*Preference

Preference*EC_total EC_total INTERCEPT=YES

DISTRIBUTION=NORMAL LINK=IDENTITY

/CRITERIA SCALE=MLE PCONVERGE=1E-006(ABSOLUTE) SINGULAR=1E-012 ANALYSISTYPE=3(WALD) CILEVEL=95

LIKELIHOOD=FULL

/EMMEANS TABLES=Condition SCALE=ORIGINAL

/EMMEANS TABLES=rating_type SCALE=ORIGINAL

/EMMEANS TABLES=Preference SCALE=ORIGINAL

/EMMEANS TABLES=rating_type*Preference SCALE=ORIGINAL

/EMMEANS TABLES=Condition*Preference SCALE=ORIGINAL

/REPEATED SUBJECT=id WITHINSUBJECT=rating_type*Preference SORT=YES CORRTYPE=INDEPENDENT

ADJUSTCORR=YES COVB=ROBUST

/MISSING CLASSMISSING=EXCLUDE

/PRINT CPS DESCRIPTIVES MODELINFO FIT SUMMARY SOLUTION.

***GEE Analysis for Political Ideology***

GENLIN trans1 BY Condition rating_type Preference (ORDER=ASCENDING) WITH Political_ideology

/MODEL Condition rating_type Preference rating_type*Preference Condition*Preference

Preference*Political_ideology Political_ideology INTERCEPT=YES

DISTRIBUTION=NORMAL LINK=IDENTITY

/CRITERIA SCALE=MLE PCONVERGE=1E-006(ABSOLUTE) SINGULAR=1E-012 ANALYSISTYPE=3(WALD) CILEVEL=95

LIKELIHOOD=FULL

/EMMEANS TABLES=Condition SCALE=ORIGINAL

/EMMEANS TABLES=rating_type SCALE=ORIGINAL

/EMMEANS TABLES=Preference SCALE=ORIGINAL

/EMMEANS TABLES=rating_type*Preference SCALE=ORIGINAL

/EMMEANS TABLES=Condition*Preference SCALE=ORIGINAL

/REPEATED SUBJECT=id WITHINSUBJECT=rating_type*Preference SORT=YES CORRTYPE=INDEPENDENT

ADJUSTCORR=YES COVB=ROBUST

/MISSING CLASSMISSING=EXCLUDE

/PRINT CPS DESCRIPTIVES MODELINFO FIT SUMMARY SOLUTION.

***MANOVA with Empathy Dichotomized***

GLM Punish_attractive Compen_attractive Mixed_attarctive Keep_attractive Punish_compass

Compen_compass Mixed_cmpass Keep_compass Punish_fair Compen_fair Mixed_fair Keep_fair Punish_strong

Compen_strong Mixed_strong Keep_strong BY Condition EC_dich

/WSFACTOR=pref 4 Polynomial

/MEASURE=attract comp fair strong

/METHOD=SSTYPE(3)

/EMMEANS=TABLES(OVERALL)

/EMMEANS=TABLES(pref)

/EMMEANS=TABLES(Condition)

/EMMEANS=TABLES(EC_dich)

/EMMEANS=TABLES(Condition*EC_dich)

/EMMEANS=TABLES(Condition*pref)

/EMMEANS=TABLES(EC_dich*pref)

/EMMEANS=TABLES(Condition*EC_dich*pref)

/PRINT=DESCRIPTIVE ETASQ OPOWER HOMOGENEITY

/CRITERIA=ALPHA(.05)

/WSDESIGN=pref

/DESIGN=Condition EC_dich Condition*EC_dich.

***MANOVA with Political Ideology Dichotomized***

GLM Punish_attractive Compen_attractive Mixed_attarctive Keep_attractive Punish_compass

Compen_compass Mixed_cmpass Keep_compass Punish_fair Compen_fair Mixed_fair Keep_fair Punish_strong

Compen_strong Mixed_strong Keep_strong BY Condition Ideology_dich

/WSFACTOR=pref 4 Polynomial

/MEASURE=attract comp fair strong

/METHOD=SSTYPE(3)

/EMMEANS=TABLES(OVERALL)

/EMMEANS=TABLES(pref)

/EMMEANS=TABLES(Condition*Ideology_dich*pref)

/EMMEANS=TABLES(Condition)

/EMMEANS=TABLES(Ideology_dich)

/EMMEANS=TABLES(Condition*Ideology_dich)

/EMMEANS=TABLES(Condition*pref)

/EMMEANS=TABLES(Ideology_dich*pref)

/PRINT=DESCRIPTIVE ETASQ OPOWER HOMOGENEITY

/CRITERIA=ALPHA(.05)

/WSDESIGN=pref

/DESIGN=Condition Ideology_dich Condition*Ideology_dich.

***3.3.4. Covariance of Ratings***

Below is the syntax for these model ran in M*Plus* 8.1

***Model 1***

TITLE: CFA RSOS Study 3

DATA:

FILE IS "C:\Data\Program Files\RSOS CFA\CFSStudy3.csv";

VARIABLE:

NAMES ARE sexu id cond fairA

punat puncomp punfair punstr

comat compcomp compfair compstr

bothat bothcomp bothfair bothstr

noat nocomp nofair nostr

ideol age EC ;

USEVARIABLES punat puncomp punfair punstr

comat compcomp compfair compstr

bothat bothcomp bothfair bothstr

noat nocomp nofair nostr ;

CATEGORICAL ARE punat puncomp punfair punstr

comat compcomp compfair compstr

bothat bothcomp bothfair bothstr

noat nocomp nofair nostr ;

MISSING ARE ALL (-999);

ANALYSIS:

TYPE = GENERAL;

ESTIMATOR IS wlsmv;

ITERATIONS = 1000;

CONVERGENCE = 0.00005;

MODEL:

F1 by bothat noat comat punat;

F2 by bothcomp nocomp compcomp puncomp;

F3 by bothfair nofair compfair punfair ;

F4 by bothstr nostr compstr punstr;

OUTPUT: STANDARDIZED MODINDICES TECH1 TECH4 SAMPSTA

***Model 2***

TITLE: CFA RSOS Study 3

DATA:

FILE IS "C:\Data\Program Files\RSOS CFA\CFSStudy3.csv";

VARIABLE:

NAMES ARE sexu id cond fairA

punat puncomp punfair punstr

comat compcomp compfair compstr

bothat bothcomp bothfair bothstr

noat nocomp nofair nostr

ideol age EC ;

USEVARIABLES punat puncomp punfair punstr

comat compcomp compfair compstr

bothat bothcomp bothfair bothstr

noat nocomp nofair nostr ;

CATEGORICAL ARE punat puncomp punfair punstr

comat compcomp compfair compstr

bothat bothcomp bothfair bothstr

noat nocomp nofair nostr ;

MISSING ARE ALL (-999);

ANALYSIS:

TYPE = GENERAL;

ESTIMATOR IS wlsmv;

ITERATIONS = 1000;

CONVERGENCE = 0.00005;

MODEL:

F1 by bothat bothcomp bothfair bothstr;

F2 by noat nocomp nofair nostr;

F3 by comat compcomp compfair compstr ;

F4 by punat puncomp punfair punstr;

OUTPUT: STANDARDIZED MODINDICES TECH1 TECH4 SAMPSTAT;
